# Multidermatomal Herpes Zoster Involving All Three Branches of the Trigeminal Nerve in an Immunocompetent Adult: A Case Report

**DOI:** 10.3390/reports9020171

**Published:** 2026-05-29

**Authors:** Vasileios Petrakis, Periklis Panagopoulos, Maria Panopoulou, Dimitrios Papazoglou, Antonios Karpouzis

**Affiliations:** 1 Department of Infectious Diseases, 2nd University Department of Internal Medicine, University General Hospital Alexandroupolis, Democritus University of Thrace, 68100 Alexandroupolis, Greece; 2 University Laboratory of Microbiology, University General Hospital Alexandroupolis, Democritus University of Thrace, 68100 Alexandroupolis, Greece; 3 University Department of Dermatology, University General Hospital Alexandroupolis, Democritus University of Thrace, 68100 Alexandroupolis, Greece

**Keywords:** herpes zoster, varicella-zoster virus, trigeminal nerve, multidermatomal, immunocompetent, valacyclovir, case report

## Abstract

**Background and Clinical Significance**: Herpes zoster (HZ), caused by the reactivation of the latent Varicella-Zoster virus (VZV), typically is confined to a single dermatome in immunocompetent individuals. Thus, multidermatomal involvement, particularly simultaneous reactivation across all three branches of the trigeminal nerve, is exceedingly rare without history of immunosuppression. **Case Presentation**: We present the case of a 60-year-old immunocompetent male who presented to the Emergency Department with a two-day history of a rapidly progressive, painful vesicular eruption over the entire left side of his face, including the intraoral mucosa. Clinical evaluation, polymerase chain reaction (PCR) and serology testing confirmed VZV reactivation across the V1, V2, and V3 dermatomes. Extensive diagnostic workup, including HIV serology and whole-body computed tomography, revealed no underlying immunodeficiency or occult malignancy. The patient was treated promptly with oral valacyclovir and topical ointments, resulting in rapid crusting and healing within one week without severe complications. **Conclusions**: This case highlights that multidermatomal trigeminal HZ can occur in healthy individuals and emphasizes the importance of prompt diagnostic workup and antiviral therapy to prevent devastating ocular and neurological sequelae.

## 1. Introduction and Clinical Significance

Primary infection with the Varicella-Zoster virus (VZV) causes varicella (chickenpox), which is clinically characterized by vesicular lesions on an erythematous base presenting in varying stages of development [1]. Following primary infection, the virus establishes latency in the sensory dorsal root ganglia and cranial nerve ganglia. Reactivation of this latent virus leads to herpes zoster (shingles) [2]. The classical clinical manifestation of herpes zoster is a localized, unilateral vesicular eruption accompanied by severe burning pain or hyperesthesia, most commonly distributed within a single thoracic or lumbar dermatome [1]. While cases involving the branches of the trigeminal cranial nerve are well-documented (most notably herpes zoster ophthalmicus involving the V1 branch), simultaneous multidermatomal involvement of the trigeminal nerve is an exceptionally rare clinical entity [3]. When multidermatomal or disseminated HZ does occur, it is heavily associated with significant cellular immunodeficiency, such as in people with human immunodeficiency virus (HIV) infection, ongoing chemotherapy, or hematological malignancies [4]. Consequently, encountering a patient with HZ affecting the ophthalmic (V1), maxillary (V2), and mandibular (V3) branches simultaneously, without any underlying immunosuppressive conditions, presents a unique diagnostic and therapeutic challenge. Herein, we report a rare case of an immunocompetent 60-year-old male presenting with pan-trigeminal herpes zoster.

## 2. Case Presentation

### 2.1. Patient Information and Clinical Presentation

A 60-year-old male presented to the Emergency Department complaining of a severe burning sensation and pain (Visual Analog Scale [VAS] score of 8/10]), and a progressive cutaneous eruption on the left half of his face that had been present for two days. The patient reported that the lesions initially appeared on his left upper lip but rapidly worsened, spreading to involve the entire left side of his face within a 24 h period. The patient denied any systemic symptoms such as fever, chills, or weight loss prior to or during the eruption. Crucially, the patient had no significant past medical history. He was not taking any routine medications and denied any history of recurrent infections, recent illnesses, or clinical conditions associated with immunosuppression. The patient had no prior history of vaccination against Varicella-Zoster virus.

### 2.2. Clinical Findings

**Figure 1:**
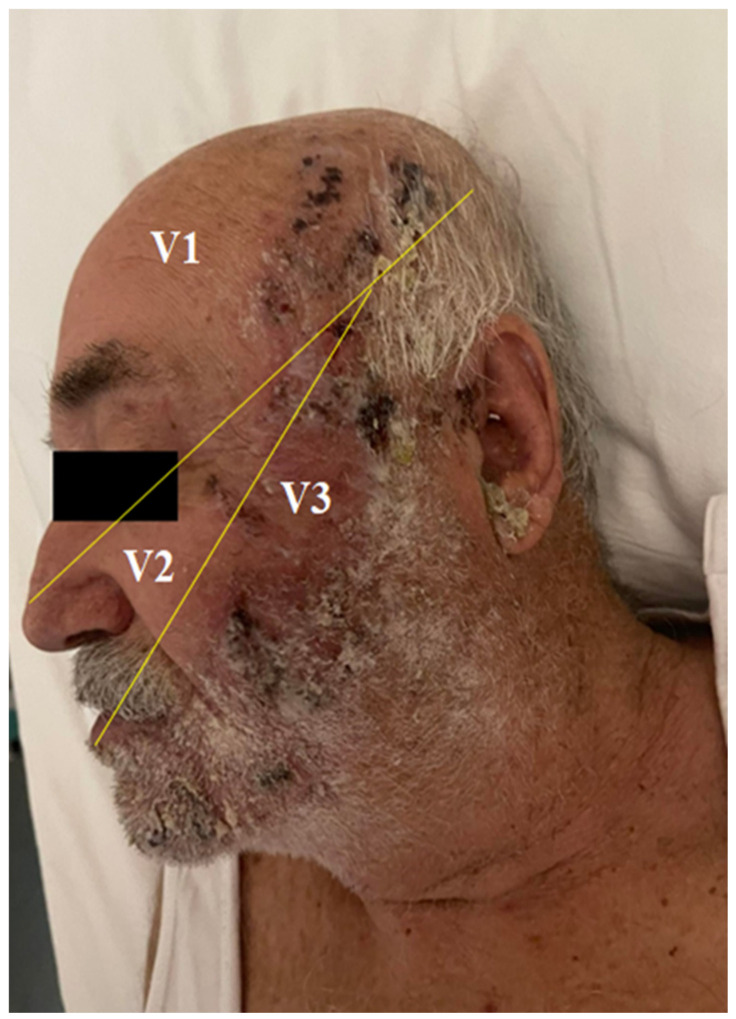
Extensive, sharply demarcated vesicular and bullous lesions distributed along the ophthalmic (V1), maxillary (V2), and mandibular (V3) divisions of the left trigeminal nerve in a 60-year-old immunocompetent male at presentation. Written informed consent for the publication of this recognizable image was obtained from the patient (Consent Document No. 001, dated 25 January 2025).

Upon physical examination, the patient’s vital signs were stable and within normal limits. Cutaneous examination revealed extensive, edematous, and sharply demarcated unilateral vesicular and bullous lesions on an erythematous base (Figure 1). The eruption strictly followed the cutaneous distribution of the left ophthalmic (V1), maxillary (V2), and mandibular (V3) branches of the trigeminal nerve. Intraoral examination further revealed the presence of corresponding vesicular lesions extending onto the left soft and hard palate. Due to the involvement of the V1 dermatome, a comprehensive ophthalmic examination of the left eye was urgently conducted. This revealed conjunctival congestion, but fortunately, there were no slit-lamp findings indicative of keratitis or uveitis. An otolaryngological evaluation was also performed due to the patient reporting mild auditory symptoms, which confirmed a mild conductive hearing loss in the ipsilateral ear, but no classical signs of herpes zoster oticus (Ramsay Hunt syndrome). The remainder of the systemic clinical examination was unremarkable.

### 2.3. Diagnostic Assessment

Given the highly atypical, multidermatomal presentation, an extensive laboratory and radiological workup was initiated to rule out occult immunodeficiency. Complete blood count, comprehensive metabolic panel, and inflammatory markers were entirely within normal limits. Serological testing for HIV was negative. Glycosylated hemoglobin (HbA1c) was 5.4%, effectively ruling out uncontrolled diabetes mellitus as a precipitating factor for viral reactivation. To definitively rule out occult malignancies—which are notorious triggers for atypical, multidermatomal zoster presentations—a whole-body computed tomography (CT) scan was performed, yielding normal results. The clinical diagnosis of herpes zoster was definitively confirmed by serological testing, which revealed positive titers of IgM and IgG VZV antibodies, as well as by polymerase chain reaction (PCR) analysis, which detected the presence of VZV DNA in swabbed smears obtained from both the cutaneous forehead lesions and the intraoral mucosa.

### 2.4. Therapeutic Intervention, Follow-Up, and Outcomes

Upon clinical suspicion of pan-trigeminal HZ, the patient was immediately commenced on antiviral therapy. He was treated with oral valacyclovir 1 g three times a day (TID) for seven days, supplemented with supportive topical ointments for local wound care and pain management. The patient exhibited a highly favorable and rapid clinical response to the antiviral regimen and was discharged. No progression of ocular involvement occurred. By the one-week follow-up appointment, the vesicular lesions had fully crusted over, and the underlying erythema and edema had resolved. The patient reported a significant reduction in acute pain (VAS score decreased to 2/10]) and did not develop immediate signs of post-herpetic neuralgia. Given the extensive cranial nerve involvement and the patient’s age, post-herpetic neuralgia (PHN) was a primary clinical concern. The patient was followed longitudinally in the outpatient clinic for one year. Throughout this 12-month follow-up period, he maintained complete resolution of the cutaneous lesions and did not develop postherpetic neuralgia, visual impairment, or any permanent neurological sequelae.

## 3. Discussion

While HZ typically presents as a unilateral vesicular eruption confined strictly to a single dermatome, more extensive skin involvement spanning several adjacent dermatomes is clinically classified as multidermatomal zoster. Conversely, spread to nonadjacent dermatomes is termed zoster duplex unilateralis or bilateralis [3]. Multidermatomal involvement is a well-documented phenomenon in patients with significant T-cell immunodeficiency—occurring up to 15 times more frequently in HIV-infected patients and in 25% of patients with Hodgkin’s lymphoma [5,6]. However, multidermatomal HZ is exceedingly rare in immunocompetent individuals. To date, a limited number (Table 1) of multidermatomal zosters in immunocompetent persons have been reported in the medical literature [7,8,9,10,11,12,13,14]. Furthermore, while the ophthalmic branch (V1) is the most commonly affected division of the trigeminal nerve, the maxillary (V2) and mandibular (V3) divisions are rarely involved.

The pathogenesis of this severe reactivation in our patient can be attributed to age-related immunosenescence. The overall incidence of HZ in the general population is approximately 5.4%, but the infection disproportionately affects individuals over 45 years of age, with the highest incidence peaking between 68 and 90 years [15]. VZV reactivation is specifically halted by robust VZV-specific cell-mediated immunity (CMI) [16]. Humoral immunity, conversely, does not appear to confer protection against viral reactivation [17]. During young adulthood, CMI is robust, but it undergoes a natural decline with advancing age, particularly crossing a critical threshold after the age of 60 [17]. This specific decline in VZV-specific CMI likely explains why our 60-year-old patient, despite lacking systemic immunosuppressive disease, was vulnerable to such an extensive, multidermatomal viral reactivation, which may have been further exacerbated by undocumented transient triggers such as physical stress or trauma. While advanced immunological assays such as CD4+/CD8+ T-cell counts or VZV-specific interferon-gamma responses can definitively quantify the extent of age-related decline in cell-mediated immunity, they are not routinely indicated in the acute clinical management of zoster absent systemic signs of severe immunodeficiency. Our workup appropriately focused on ruling out occult malignancies and HIV, aligning with standard clinical pathways.

Clinically, HZ progresses through distinct prodromal, active, and chronic stages. The classical prodrome consists of mild-to-moderate burning, tingling, or paresthesia occurring 48 to 72 h prior to the development of erythematous maculopapular rashes, often accompanied by malaise [18]. In the active stage, these rapidly evolve into grouped vesicles [18]. Anatomically, HZ involves thoracic dermatomes in 53% of cases, while cranial nerves account for 20%, of which the trigeminal nerve comprises 15% [18]. The clinical manifestations strictly mirror the affected neural branches. Vesicles on the tip or side of the nose (Hutchinson’s sign) indicate involvement of the nasociliary branch of the V1 division, which correlates with ocular involvement in 30–40% of patients [19]. Involvement of the V2 division yields vesicles on the uvula and tonsillar area, while V3 involvement manifests on the anterior tongue, floor of the mouth, and buccal mucosa [20]. Our patient exhibited classic cutaneous lesions across all three dermatomes, alongside the corresponding intraoral manifestations extending to the soft and hard palate.

In multidermatomal cranial HZ, the primary clinical imperative is to prevent irreversible ocular and neurological complications [21]. In immunocompetent patients, zoster is typically a self-limiting disease, but it frequently leads to severe morbidity [7,8,9,10,11,12,13,14]. Among these, postherpetic neuralgia (PHN) is the most common, affecting 10–20% of patients and characterized by deep, brief, recurrent shooting allodynia that can last for months or years [22]. Furthermore, cranial involvement carries a high risk of catastrophic ocular sequelae, including subconjunctival hemorrhage, epithelial and stromal keratitis, anterior uveitis, optic neuritis, oculomotor nerve palsy, and retinal necrosis [23]. Other potential complications include secondary bacterial infections (e.g., group A β-hemolytic streptococcal cellulitis), peripheral nerve palsies, Ramsay Hunt syndrome (if the geniculate ganglion is involved), and rare dental complications such as root resorption or alveolar osteonecrosis [24]. Given these risks, our patient’s prompt ophthalmological and otolaryngological evaluations were critical, fortunately revealing only conjunctival congestion and mild conductive hearing loss without destructive keratitis or permanent facial paralysis.

Accurate and rapid diagnosis is paramount to minimizing these complications. Although clinical diagnosis of HZ is common in typical presentations, the atypical, disseminated nature of multidermatomal zoster often necessitates laboratory confirmation [25]. The initial differential diagnosis for extensive facial vesiculobullous eruptions includes severe Herpes Simplex Virus (HSV) infection, multifocal impetigo or bacterial cellulitis, and autoimmune bullous dermatoses such as pemphigus vulgaris or bullous pemphigoid. However, the strict unilateral, multidermatomal distribution heavily favored VZV, which was subsequently confirmed. Traditionally, the Tzanck smear assay is utilized to identify multinucleated giant cells and intranuclear inclusion bodies [25]. However, this method has low sensitivity, is highly dependent on the presence of fresh vesicles, and cannot differentiate between VZV and herpes simplex virus (HSV-1/HSV-2) [25]. Viral culture, while definitive, is labor-intensive and time-consuming [26]. Serological testing can confirm acute infection, with IgM antibodies appearing within 5 days of the rash and IgG indicating past exposure [26]. Today, the gold standards for rapid, highly specific diagnosis in complex presentations are direct fluorescent antibody (DFA) testing and Polymerase Chain Reaction (PCR) [27]. PCR is considered the gold standard, offering a sensitivity and specificity approaching 100%, far surpassing the Tzanck smear, which has a sensitivity of merely 50–60% and cannot distinguish between VZV and HSV [25,26,27]. In our case, the prompt utilization of PCR from mucosal smears provided definitive confirmation of VZV DNA, bypassing the limitations of traditional cytology.

Finally, early institution of antiviral and symptomatic therapy significantly reduces overall morbidity [28]. Viral replication largely ceases 72 h after the onset of the rash; therefore, systemic antivirals must be initiated within this critical window [28]. Guanosine analogues, such as acyclovir (800 mg, five times daily) or valacyclovir (1000 mg, three times daily), are selectively phosphorylated by viral thymidine kinase, effectively inhibiting viral DNA polymerase [28]. Valacyclovir, used in our case, is a prodrug that achieves serum acyclovir levels three to five times higher than oral acyclovir therapy, shortening viral shedding and hastening healing [29]. While the concomitant use of systemic corticosteroids can accelerate cutaneous healing and alleviate acute pain, they remain controversial due to the risk of exacerbating immunosuppression and have not been shown to decrease the incidence of PHN [30]. Consequently, they were omitted from our treatment regimen. Due to the immediate administration of valacyclovir, our patient exhibited a rapid response, with complete crusting and healing within a week.

## 4. Conclusions

Multidermatomal herpes zoster involving all three trigeminal branches is a rare clinical manifestation in an immunocompetent patient. This case demonstrates that while such an aggressive presentation should prompt an immediate and thorough investigation to rule out hidden immunodeficiency or malignancy, it can occur in healthy individuals. Maintaining a high index of clinical suspicion and ensuring the immediate initiation of systemic antiviral therapy is paramount in preventing severe, permanent ocular and neurological complications.

## Figures and Tables

**Table 1:** Review of reported cases of multidermatomal herpes zoster in immunocompetent patients.

	Patient (Age, Gender)	Affected Dermatomes	Clinical Manifestations at Admission	Treatment, Complications, Clinical Outcome
Nair P et al. [7]	68 y, Male	V2–V3	• No relevant medical, dental or family history • Blisters on the entire left half of the face with a watery discharge. • Fever for 5 days	• Valacyclovir 1 g × 3 (1 week) • Tramadol hydrochloride (100 mg), acetaminophen (325 mg) • Ramsay-Hunt syndrome, Bell’s palsy • Complete regression after 2 weeks
Nair P et al. [7]	50 y, Male	V1–V3	• No medical history • Fever, blisters and pain on the left side of the face for the past 2 days. • Very severe, burning, continuous pain	• Acyclovir 800 mg × 5 (1 week) • Tramadol hydrochloride (100 mg), acetaminophen (325 mg) • Complete regression after 2 weeks
Naveen KN et al. [8]	28 y, Male	V1–V3	• No medical history • Multiple fluid filled lesions over the left half of the face for 3 days. • Burning sensation and pain over the lesions.	• Conductive hearing loss and conjunctival congestion. • Acyclovir 800 mg × 5 (1 week) • Topical antibiotics • Complete regression after 5 days
Dube S et al. [9]	55 y, Male	V1–V3	• No medical history • Pustular, vesicular, and maculopapular eruptions over the right half of face.	• Conjunctival congestion. • Acyclovir 800 mg × 5 (2 weeks) • Diclofenac 100 mg × 2 • Recovery within a week
Srivastava RM et al. [10]	55 y, Female	V1–V2	• Redness, pain, watering, and blurring of vision in the right eye • Vesicular eruptions over the right half of the face and buccal mucosa for 1 day. • History of an accidental trauma to the skin of the right cheek and nose with a wooden stick 3 days ago.	• Topical eye drops moxifloxacin 0.5% and carboxymethylcellulose • Oral acyclovir 800 mg 5 times a day (7 days) • Cefuroxime 200 mg b.d, and multivitamins for 7 days. • Recovery after 1 week
Pelloni LS et al. [11]	71 y, Male	V1–V3	• Grouped vesicular lesions with crusts on the left half of the face of two-weeks duration. • Unremarkable past medical history.	• Conjunctival congestion in the left eye with lid edema • Oral valaciclovir 1 g ×3, oral paracetamol and topical fucidic acid ointments • Complete recovery within 10 days
Jung T et al. [12]	35 y, Female	V2–V3	• Painful erythematous vesicles, pustules, and scabs of various stages on her right neck, cheek, scalp, and mandibular area (for 7 days) • Rotatory vertigo and right-sided hearing loss with tinnitus	• Acute vestibulocochlear deficit • Systemic steroids, antiviral agents, vestibular suppressants with antiemetics, intratympanic steroid injection for acute sensorineural hearing loss. • Gradual complete recovery
Sun CE et al. [13]	29 y, Female	V2–V3	• Vesicular lesions with crusts on left side of face for 3 days associated with left sided facial pain and burning sensation • No medical history	• Acyclovir for 10 days, topical Acyclovir cream, mouth rinse and oral analgesics. • Complete recovery within 2 weeks
Sundar S et al. [14]	27 y, Male	V1–V3	• High-grade fever for 1 week, followed by multiple, painful rashes on the face and painful oral ulcers. • No medical history	• Valacyclovir 1 g ×1 (1 week) • IV antibiotics (Cefotaxime 1 g ×3, Clindamycin 600 mg ×3) and oral antifungals (Fluconazole 100 mg ×1) • Topical moisturizers and pregabalin 75 mg ×2 • Recovery after 1 week

## Data Availability

The research data are available after applying to the corresponding author due to privacy concerns.
